# The Role of Renalase in Cardiovascular Disease: A Comprehensive Review of Its Molecular Biology, Genetic Associations, and Clinical Significance

**DOI:** 10.3390/life15101581

**Published:** 2025-10-10

**Authors:** Siarhei A. Dabravolski, Andrey V. Omelchenko, Elizaveta R. Korchagina, Olga N. Maltseva, Vsevolod V. Pavshintsev, Tatiana I. Kovyanova

**Affiliations:** 1 Department of Biotechnology Engineering, Braude Academic College of Engineering, Snunit 51, P.O. Box 78, Karmiel 2161002, Israel; 2 Institute for Atherosclerosis Research, Osennyaya Street 4-1-207, 121609 Moscow, Russia; omi@bk.ru (A.V.O.); lizavetacorchagina@yandex.ru (E.R.K.); 3 Institute of Experimental Medicine, 12 Academician Pavlov Street Street, 197022 Saint Petersburg, Russia; movolya@mail.ru; 4 Institute of Ecology, Peoples’ Friendship University of Russia (RUDN University), 6 Miklukho-Maklaya Street, 117198 Moscow, Russia; vsevolodpav@mail.ru; 5 Institute of General Pathology and Pathophysiology, 8 Baltiyskaya Street, 125315 Moscow, Russia; kovyanovat@gmail.com

**Keywords:** renalase, cardiovascular disease, biomarker, gene polymorphism, hypertension, heart failure

## Abstract

Renalase, a flavin adenine dinucleotide (FAD)-dependent enzyme/hormone, has emerged as a molecule of significant interest in cardiovascular medicine since its discovery nearly two decades ago. Initially proposed as a catecholamine-degrading enzyme crucial for blood pressure regulation, its functional repertoire has expanded to include potential α-NAD(P)H oxidase/anomerase activity and roles as a signalling cytokine. This review synthesises the current understanding of renalase, encompassing its fundamental biology, intricate gene regulation by transcriptional factors, microRNAs, and physiological stimuli, and its implications in cardiovascular health and disease. A central focus is the critical appraisal of circulating renalase as a clinical biomarker. We critically evaluate findings from preclinical animal and cellular models related to atherosclerosis, heart failure, and blood pressure control. Furthermore, this review examines the extensive literature on RNLS gene polymorphisms and their associations with human cardiovascular phenotypes, alongside the complex and often context-dependent data regarding circulating renalase levels as a potential clinical biomarker in conditions such as hypertension, coronary artery disease, atrial fibrillation, and heart failure. While renalase shows context-specific promise, its multifaceted biology and the current methodological disparities limit its immediate clinical application. This review concludes by outlining a clear path forward, emphasising the need for standardised research and mechanistic studies to unlock the true diagnostic and therapeutic potential of renalase in CVD.

## 1. Introduction

Globally, cardiovascular disease (CVD) represents a substantial health challenge, contributing significantly to disability, early death, and escalating healthcare expenditures [1,2]. In 2019 alone, CVD was responsible for 6.2 million fatalities among individuals aged 30 to 70 years. Furthermore, between 1990 and 2019, the number of prevalent CVD cases surged from 271 million to 523 million, a near doubling, while associated deaths climbed from 12.1 million to 18.6 million [3]. A particularly concerning trend is the observation that age-standardised CVD rates, which had been on a downward trend in high-income nations, have started to increase. Concurrently, the impact of CVD is intensifying across most populations beyond high-income regions [4], and projections suggest an annual death toll of 24 million by 2030 [2], with the condition consuming 11% of total healthcare expenditure in Europe [5]. Even with the application of evidence-based treatments, improvements in patient survival and quality of life over the past two decades have been limited [6]. Consequently, there is an urgent need to supplement current therapeutic strategies, which primarily address neurohumoral over-activation, by exploring the fundamental intrinsic mechanisms that drive the onset and progression of CVD [7].

**Figure 1:**
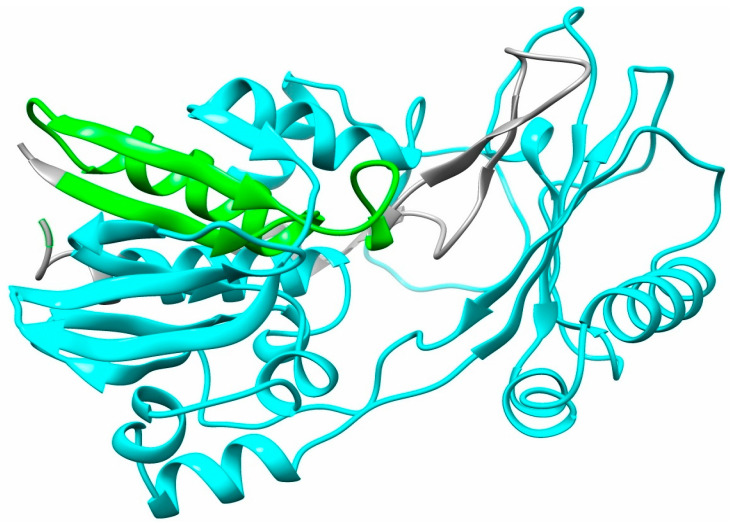
Three-dimensional structure of human renalase isoform 1. The ribbon diagram is based on the AlphaFold predicted structure (UniProt: Q5VYX0). The key functional domains are highlighted: the FAD-binding domain (amino acids 3–42) is shown in green, and the amine oxidase domain (amino acids 75–335) is shown in cyan. The remaining regions are colored grey.

The renalase gene was initially brought to light by the Desir laboratory at Yale School of Medicine in 2005 [8], following its identification within the Mammalian Gene Collection project archives [9]. This discovery stemmed from a search for renal secretory proteins characterised by a signal peptide sequence, an absence of transmembrane domains, and sequence homology below 20% with known proteins. Termed renalase due to its discovery context, this novel enzyme/hormone is a protein of 342 amino acids, with an estimated mass of approximately 38 kDa, and its primary structure shows partial similarity to monoamine oxidases. Based on its catalytic activity’s dependence on flavin adenine dinucleotide (FAD), renalase was categorised as a new FAD-containing amine oxidase [8]. It has since been described using multiple terms reflecting its diverse proposed functions: an ‘enzyme’ for its FAD-dependent catalytic potential, a ‘hormone’ as it is secreted into circulation to act on distant sites, and ‘cytokine-like’ due to its ability to function as an extracellular signalling molecule [10,11]. Despite early suppositions of a predominantly renal origin, renalase has since been found in tissues such as the myocardium, adipose tissue, liver, both peripheral and central nervous systems, the small intestine, and skeletal muscles [12,13]. Positioned on chromosome 10 at locus q23.31, the gene comprises ten exons [14] and extends over roughly 311,000 base pairs [8]. While seven isoforms of the human renalase gene have been identified (renalase 1–7), isoform 1 is the most abundant and is considered responsible for the majority of its biological functions [15,16]. Renalase isoform 1 incorporates a signal peptide (amino acids 1–17), a region for FAD binding (amino acids 3–42), and an amine oxidase domain (amino acids 75–335) (Figure 1).

The unveiling of renalase triggered a surge in research activity, fuelled by its compelling scientific implications [17,18,19]. The initial hypothesis posited that renalase enzymatically degrades catecholamines, favouring dopamine as a substrate, and thereby influences cardiovascular dynamics by lowering blood pressure and heart rate [8]. However, this proposed function became a subject of significant controversy, as subsequent studies using highly purified recombinant renalase failed to detect significant monoamine oxidase activity towards catecholamines or other biogenic amines [20,21], directly challenging the conclusions of the original discovery [8]. This debate prompted a shift in focus towards alternative functions. A new perspective was introduced by Graham H. Moran’s group, who demonstrated renalase’s capacity to function as an α-NAD(P)H oxidase/anomerase, oxidising inhibitory isomers of β-NAD(P)H (2- and 6-dihydroNAD(P)) to generate biologically active β-NAD(P)^+^ and H_2_O_2_ [18,22]. These findings suggest a more relevant intracellular or metabolic role for renalase, potentially supporting normal respiratory processes. Concurrently, evidence has emerged for a non-catalytic, “moonlighting” characteristic of renalase as a signalling molecule. Acting akin to a cytokine, extracellular renalase can engage with a plasma membrane calcium ATPase isoform, PMCA4b (the renalase receptor), to activate downstream signalling cascades, thereby exerting various biological effects, including cytoprotection [10,11]. More recently, this signalling function has also been implicated as a survival factor in some cancers, potentially promoting tumour development [23,24,25].

The early conceptual “renalase pathway” attempted to integrate the catecholamine hypothesis with blood pressure regulation, suggesting that circulating inactive “prorenalase” is activated by surges in catecholamines or blood pressure, leading to catecholamine degradation in a negative feedback loop [14,26]. This framework also proposed that renalase could decrease cardiac contractility and heart rate, inducing hypotension [8,27]. However, accumulating evidence refuting the primary catecholamine-metabolising capability of renalase, such as the work by Beaupre et al. [20], has cast considerable doubt on the validity of this pathway as originally described.

In this review, we aim to provide a thorough examination of the multifaceted role of renalase in the context of cardiovascular disease. We will begin by revisiting its fundamental biological characteristics, including its discovery, structural features, and the ongoing discourse surrounding its enzymatic activities and signalling pathways. Subsequently, we will delve into the current understanding of renalase gene expression regulation, exploring transcriptional, post-transcriptional, and physiological modulators. The review will then synthesise findings from preclinical research, discussing evidence derived from cellular and animal models concerning renalase’s involvement in blood pressure control and specific cardiovascular pathologies such as atherosclerosis and heart failure. Transitioning to human studies, we will critically assess the literature on renalase gene polymorphisms (SNPs) and their reported associations with various cardiovascular phenotypes and disease risks. Finally, we will evaluate the extensive data on circulating renalase levels as a potential clinical biomarker across a spectrum of conditions, including hypertension, coronary artery disease, atrial fibrillation, and heart failure, also considering its correlation with broader cardiovascular risk factors and outcomes. By consolidating these diverse research avenues, this review seeks to offer a contemporary perspective on the intricate involvement of renalase in cardiovascular health and disease, highlighting both established knowledge and areas necessitating further elucidation to fully understand its diagnostic and therapeutic potential.

## 2. Molecular and Physiological Regulation of Renalase Gene Expression

Substantial research has explored the biochemistry and physiology of renalase since its identification; however, a comprehensive understanding of the molecular mechanisms governing renalase gene expression remains comparatively underdeveloped. At the transcriptional level, investigations have recently pinpointed the transcription factors Sp1, STAT3, and ZBP89 as pivotal molecular regulators of human renalase gene expression [28]. Beyond basal control, renalase expression is also highly responsive to cellular stress signals. In the context of hypoxia, it has been identified as a hypoxia-responsive gene in cardiomyocytes, where hypoxia-inducible factor-1α (HIF-1α) drives its expression, thereby affording protection against myocardial ischaemia–reperfusion injury [29]. A similar protective role is observed in the kidneys, where HIF1α-mediated induction of renalase expression aids in renoprotection conferred by ischaemia preconditioning [30]. Inflammatory and hormonal stimuli also play a role; in fatty liver conditions, for instance, diminished renalase expression correlates with reduced STAT3 levels [31], while epinephrine-triggered expression in proximal tubular epithelial cells is mediated by the α-adrenoceptor/NF-κB pathway [32]. Furthermore, NFκB-driven upregulation of renalase expression in the intestine offers protection from fasting-induced oxidative stress [33]. In addition to transcriptional control, renalase is subject to post-transcriptional regulation by microRNAs, including miR-29b and miR-146a [34]. Clinically, low miRNA-146a expression alongside high renalase levels has been associated with longer survival times in haemodialysis patients [35]. Another study in hypertensive subjects found that renalase levels negatively correlated with miRNA-4516 and positively with miRNA-145, both of which were identified as independent predictors of hypertension [36].

**Figure 2:**
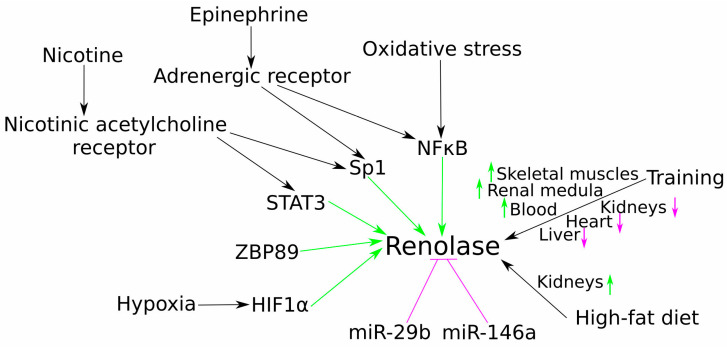
Key regulators of renalase gene expression. Schematic overview of known molecular and physiological factors modulating renalase expression. Transcriptional regulation is mediated by basal (e.g., Sp1, ZBP89) and stimulus-responsive (e.g., STAT3, HIF-1α, NF-κB) transcription factors. Post-transcriptional control is exerted by microRNAs (e.g., miR-29b, miR-146a). Physiological stimuli such as exercise and diet also induce complex, tissue-specific changes in renalase levels. Abbreviations: hypoxia-inducible factor-1-alpha (HIF-1α), ischaemic preconditioning (IPC), ischaemia–reperfusion (IR), zinc-binding protein 89 (ZBP89), signal transducer and activator of transcription 3 (STAT3), specificity protein 1 (SP1), nuclear factor kappa B (NFκB), microRNA (miR).

The impact of physiological stimuli on renalase regulation has been further explored in various experimental models. The effects of physical exercise, in particular, reveal complex, tissue-specific responses. Experiments on spontaneously hypertensive rats (SHRs) showed that 14 weeks of moderate training improved renal function and increased renalase levels in the renal medulla. This protective effect may be linked to the observation that, in vitro, renalase inhibited Ang II-induced apoptosis and oxidative stress in HK-2 kidney cells [37]. In another study on Wistar rats subjected to acute treadmill exercise, renalase expression increased in skeletal muscles, while it was reduced in the kidneys, heart, and liver. This study further established that NF-κB and HIF-1α were responsible for regulating renalase expression in the plantaris and soleus muscles, respectively, suggesting that renalase may play a protective role in preventing exercise-induced oxidative stress [38]. A subsequent study found that renalase levels in blood were increased after moderate treadmill exercise in Wistar rats. Particularly, renalase expression in the kidney was decreased after exercise, while it was increased in the extensor digitorum longus and plantaris muscles. Interestingly, renalase expression in other tissues was not affected [39]. Metabolic factors also modulate renalase; further experiments showed that both a high-fat diet and exercise increased its gene expression in the kidneys, whereas in skeletal muscles, the increase was driven only by exercise training [40] (Figure 2).

Taken together, these studies illustrate that renalase gene expression is under intricate control, influenced by a diverse array of transcription factors, microRNAs, and physiological stimuli including hypoxia, hormonal signals, exercise, and diet. This complex regulatory network likely dictates tissue-specific renalase levels and their responsiveness to various pathological conditions. A more thorough understanding of renalase gene regulation, particularly the interplay between these factors under both basal and pathophysiological states, is crucial for fully elucidating its role in cardiovascular health and disease, and necessitates further dedicated mechanistic studies employing both in vitro and in vivo models.

## 3. Renalase and Its Association with Blood Pressure Regulation

Much of the initial interest in renalase stemmed from its proposed role in blood pressure regulation, a concept built upon the hypothesis that it functions as a circulating catecholamine-degrading enzyme. This idea was first highlighted by Xu et al. [8], whose findings demonstrated that administering recombinant renalase led to reductions in systolic, diastolic, and mean arterial pressure. Their in vitro experiments suggested that renalase could metabolise vasoactive catecholamines, such as dopamine, epinephrine, and norepinephrine, leading to the speculation that its hypotensive effects were mediated by catecholamine degradation. This spurred considerable research and led to the formulation of a conceptual “renalase pathway.” This early framework proposed that inactive circulating “prorenalase” could be activated by surges in blood pressure or catecholamines, after which it would metabolise these amines in a negative feedback loop, reduce cardiac contractility, and lower blood pressure in a manner resembling nonselective β-adrenoceptor antagonism [8,26,41,42]. However, as noted in the introduction, this specific mechanism has since been widely challenged.

### Mechanistic Insights from Preclinical Models

Evidence supporting a crucial role for renalase in blood pressure homeostasis comes from various genetic and interventional animal models, which consistently show that renalase deficiency or suppression leads to hypertension. For instance, global renalase knockout (KO) mice, which are deficient in the promoter region and a substantial portion of the renalase gene’s coding sequence [43], exhibited tachycardia and elevated blood pressure, particularly diastolic pressure. This finding suggests significant vasoconstriction, likely stemming from sympathetic activation due to increased systemic catecholamine levels in the absence of renalase. Similarly, studies employing salt-sensitive Dahl rats fed an 8% salt diet, a recognised model for investigating hypertension mechanisms, have clearly shown that these animals develop moderately severe hypertension without evident renal dysfunction, an outcome attributed to an early onset of renalase deficiency [44]. Some investigations propose that renalase might modulate blood pressure and sympathetic tone by influencing the adrenergic system’s pressor response. In rats treated with renalase antisense RNA, diminished renalase levels were associated with increased systolic blood pressure and a nearly doubled norepinephrine-mediated pressor effect [26]. Conversely, subcutaneous delivery of recombinant renalase in 5/6 nephrectomised (Nx) rats notably lowered blood pressure without altering heart rate [27].

Given the known interplay between the sympathetic nervous system and dopaminergic receptors in blood pressure regulation, the impact of renalase on dopamine metabolism has also garnered significant research attention [45]. The crucial role of the renal dopamine system in sodium and phosphate balance, combined with the observation that sodium overload inhibits renalase expression in animals, suggested that renalase could influence blood pressure via the dopamine system [44]. Studies with renalase knockout mice indicate that renalase deficiency increased urinary phosphate excretion through a mechanism involving heightened renal dopamine synthesis [46]. Moreover, renalase-deficient conditions lead to an upregulation of the L-DOPA transporter LAT1, increasing L-DOPA uptake and activating the renal dopaminergic system, an effect reversed by external renalase administration [47]. The expression of renalase itself appears to be modulated by dopaminergic signalling, as the D5 receptor agonist fenoldopam showed opposing effects on renalase expression in normotensive versus spontaneously hypertensive rats [48], suggesting a complex feedback loop between the dopaminergic system and renalase in the context of catecholamine metabolism [49].

Further evidence linking renalase to sympathetic nervous system activity comes from studies on renal denervation, a non-pharmacological approach to lower blood pressure by ablating renal sympathetic nerves [50]. Research by Jiang et al. [51] provided initial evidence that renal denervation in spontaneously hypertensive rats led to a marked decrease in mean arterial pressure accompanied by a significant upregulation of renalase expression. These results imply that the blood pressure-lowering effect of renal denervation might be mediated through increased renalase expression.

In summary, as highlighted in a recent review [52], these collective findings suggest that renalase may influence blood pressure through several mechanisms: (1) metabolising catecholamines; (2) inhibiting renal dopamine activation; (3) reducing renal sympathetic nervous system activity. However, it is pertinent to note that due to considerable, well-founded scepticism regarding some of these proposed mechanisms, researchers in the renalase field have introduced a newer hypothesis. This posits that renalase might affect blood pressure and vascular tone via its α-NAD(P)H oxidase/anomerase activity [22]. In this model, the hypotensive effects observed with recombinant renalase could be explained not by catecholamine removal, but by the generation of vasodilatory signalling molecules such as H_2_O_2_ from the oxidation of isomeric forms of β-NAD(P)H [17,53]. While this concept presents an appealing alternative, further evidence-based studies are necessary to substantiate this proposed mechanism.

## 4. Renalase in Preclinical Cardiovascular Disease Models

Investigating the role of renalase in preclinical models of cardiovascular disease provides crucial insights into its potential mechanisms of action and therapeutic relevance. Studies utilising various animal and cellular systems have begun to explore renalase’s involvement in distinct pathological processes, including atherosclerosis and adverse cardiac remodelling.

The involvement of renalase has been implicated in atherosclerosis. Recent research by Zhou et al. [54] demonstrated that valsartan, an angiotensin II type 1 (AT1) receptor antagonist, stabilised atherosclerotic plaques in *ApoE*^−/−^ high-fat-fed mice, an effect at least partially mediated through renalase. The study noted complex, tissue-specific changes in renalase expression in this model: compared to wild-type control mice, renalase expression was increased in the fat tissue and brain of *ApoE*^−/−^ mice. Furthermore, in high-fat-fed *ApoE*^−/−^ mice, renalase expression was elevated in the kidney, testes, and brain, while its expression in the liver was decreased relative to wild-type controls on a standard diet. Within established atherosclerotic plaques, renalase expression was detected in smooth muscle cells and M2 macrophages; however, in the fibrous cap, its expression gradually decreased during long-term high-fat feeding. Correspondingly, valsartan treatment increased serum renalase levels and its expression in the fibrous cap of the atherosclerotic plaque. Additionally, valsartan increased the expression of Arg-1 and α-actin in the fibrous caps, enhanced renalase expression in the liver, and reduced serum triglyceride levels. Collectively, these results suggest that valsartan may improve lipid metabolism and stabilise atherosclerotic plaques by influencing renalase expression [54].

Beyond atherosclerosis, renalase has also been extensively studied in the context of adverse cardiac remodelling, including left ventricular hypertrophy (LVH) and heart failure (HF). In a rat model of pressure overload-induced LVH and heart failure, renalase levels exhibited a biphasic response, initially increasing during LVH development and subsequently decreasing with the progression of heart failure. This dynamic expression pattern suggests a potentially adaptive initial role followed by maladaptive depletion. Mechanistically, inhibition of renalase using siRNA downregulated p-P38 and p-ERK1/2 signalling, alleviated noradrenaline-induced hypertrophy in an in vitro cell model, and diminished the pressure overload-induced hypertrophic response in vivo. Conversely, administration of recombinant renalase improved cardiac function in rats with HF by inhibiting cardiac fibrosis, as evidenced by reduced levels of cardiac fibrosis markers collagen I and III. These findings pointed to renalase as a potential target in p38 and ERK1/2-mediated therapeutic approaches for HF [55].

The cardioprotective and anti-fibrotic potential of renalase is further supported by studies in models of chronic kidney disease (CKD)-induced pathological cardiac remodelling. In a mouse model, renalase knockout exacerbated such remodelling, whereas cardiac-specific renalase overexpression attenuated LVH and cardiac fibrosis induced by CKD. Elucidating the underlying mechanisms, subsequent RNA-seq analysis revealed that renalase overexpression downregulated several extracellular matrix (ECM) pathway-related genes, including those for various collagens (*COL1A1/2*, *COL3A1*, *COL4A1/2*). Additionally, renalase overexpression suppressed pro-fibrotic processes, such as epithelial–mesenchymal transition (EMT), antigen processing and presentation, migration of immune cells, fibroblast proliferation, and cytokine production, which were upregulated by CKD. Corroborating these in vivo findings, in vitro cultures of primary cardiac fibroblasts showed that renalase overexpression reduced TGFβ-stimulated proliferation and *αSMA* expression. These comprehensive genetic studies provide strong evidence for a direct, protective role of cardiac-derived renalase against the adverse remodelling seen in cardiorenal syndromes [56].

In summary, these preclinical investigations highlight diverse and sometimes complex roles for renalase in cardiovascular pathologies. Evidence from atherosclerosis models implicates it in plaque dynamics and lipid metabolism, while studies on cardiac remodelling and heart failure consistently point towards a protective, anti-fibrotic function, particularly in the context of pressure overload and cardiorenal stress. While its expression and actions can vary depending on the specific disease model and stage, evidence suggests that modulating renalase activity or expression may hold therapeutic potential for conditions like atherosclerosis and heart failure, particularly by influencing inflammation, fibrosis, and key signalling pathways. Further preclinical work is warranted to delineate these mechanisms more precisely and to assess the translatability of these findings to human CVD.

## 5. Renalase Gene Polymorphisms and Their Association with Cardiovascular Diseases and Related Phenotypes

Genetic variations within the *RNLS* gene, which encodes renalase, have been extensively investigated for their potential associations with CVDs and related intermediate phenotypes. Understanding these single-nucleotide polymorphisms (SNPs) could offer insights into disease susceptibility, progression, and inter-individual variability in cardiovascular health. However, it is crucial to interpret these findings with caution, as many studies are conducted in specific ethnic populations with modest sample sizes, and inconsistent replication across cohorts is a common challenge. This section reviews key studies examining the links between specific *RNLS* gene variants and various cardiovascular parameters (Table 1).

One notable functional polymorphism, Glu37Asp (rs2296545), has been linked to structural cardiac changes. For instance, in a cohort of elderly Polish patients with aortic stenosis, the Asp37 variant was associated with increased left ventricular mass, intraventricular septal thickness, posterior wall thickness, and relative wall thickness, although these effects were observed exclusively in female patients. No such associations were found among male patients, suggesting a sex-specific influence of this polymorphism on left ventricular hypertrophy in the context of aortic stenosis [57]. The rs2296545 SNP has also been explored in other conditions. Among middle-aged Egyptian patients with chronic kidney disease (CKD), the CC genotype and C allele of this SNP were linked to an increased risk of CKD itself and a higher propensity for developing hypertensive CKD [59]. Further highlighting its relevance to hypertension, in middle-aged Chinese Han patients with obstructive sleep apnoea (OSA), the CG genotype of rs2296545 was associated with an increased hypertension risk specifically within the severe OSA group. This study also noted complex relationships with serum renalase levels, which were higher in severe OSA patients and showed differential correlations with blood pressure depending on OSA status [58].

The intronic SNP rs10887800 has also garnered considerable attention, yielding varied results across different populations and clinical settings. In a study involving elderly Iranian patients with CAD and hypertension who were receiving multiple cardiovascular medications, rs10887800 showed no significant association with the risk of, or protection against, hypertension or CAD. Interestingly, this study did find higher renalase activity in patients with hypertension alone and noted differential effects of atorvastatin and losartan on this activity, suggesting a complex interplay between genetics, disease state, and pharmacological interventions in this south-east Iranian population [60]. However, in a different cohort of elderly Iranian patients presenting with MetS and unstable angina pectoris (USAP), the AG and GG genotypes of rs10887800 were more prevalent compared to controls. This finding, coupled with higher circulating renalase levels in the USAP + MetS patients, implied an association between these genotypes and an increased risk for this combined cardiometabolic condition [61]. Further linking rs10887800 to vascular events, an investigation in middle-aged Iranian patients with ischaemic stroke (IS) revealed that the AG genotype was associated with a 1.6-fold higher risk of IS [62].

Another frequently studied SNP, rs2576178, also located in an intronic region, has shown inconsistent associations. While the aforementioned Iranian ischaemic stroke study found no significant associations for rs2576178 [62], contrasting research in an elderly Chinese CAD cohort demonstrated that the GG genotype or G allele of rs2576178 was indeed associated with an increased risk of CAD. This risk appeared to be particularly pronounced among females, smokers, and individuals who consume alcohol, though no link was found with general clinical parameters in the broader CAD group [63].

The influence of renalase gene variants on blood pressure regulation extends to salt sensitivity. In a study of middle-aged American hypertensive patients, a specific haplotype (-TCTTAGTT) composed of seven *RNLS* SNPs (rs1932531, rs77324767, rs10749565, JHU_10.89888367, rs77287889, rs868872, and rs10509536) was associated with systolic blood pressure, pulse pressure, and mean arterial pressure under a high-salt diet. Moreover, carriers of the rs10887801 TT genotype exhibited higher salt sensitivity of blood pressure (SSBP) and lower plasma renin activity, supporting renalase’s role in SSBP pathogenesis and identifying individuals potentially at greater risk of hypertension from high dietary salt intake [64].

Longitudinal studies provide valuable insights into the impact of *RNLS* SNPs over time. A 14-year follow-up of middle-aged and elderly Chinese hypertensive patients identified several associations: rs7922058 was linked to changes in systolic BP; rs1935582 and rs2576178 were associated with changes in mean arterial pressure; and a broader set of SNPs, including rs2576178, rs1935582, rs10887800, rs796945, and rs2296545, were connected to changes in diastolic BP. Furthermore, rs1935582, rs2576178, and rs796945 were associated with the incidence of new-onset hypertension. Adding a molecular dimension, this study also found that *RNLS* gene expression in renal biopsy samples was decreased in hypertensive patients compared to normotensive controls, further underscoring renalase’s potential involvement in hypertension development [65].

In summary, investigations into *RNLS* gene polymorphisms have revealed several associations with structural heart changes, hypertension risk, CAD, stroke, and metabolic parameters. However, as noted, these findings are frequently population-specific and display inconsistent replication across different ethnic groups and clinical contexts. For instance, the associations for prominent SNPs like rs10887800 and rs2576178 vary significantly between the Iranian and Chinese cohorts reviewed here. This highlights the complexity of genetic contributions to CVD, which are likely influenced by strong gene–environment interactions that differ between populations.

A significant limitation of the current body of literature is the risk of overinterpreting these association studies. The majority of the reviewed studies establish statistical correlations rather than causal relationships. To bridge this gap, future research must prioritise functional validation to determine how these disease-linked SNPs concretely impact renalase biology: for example, by altering gene expression, protein stability, enzymatic activity, or signalling capacity. Without such functional data, the translational potential of these genetic findings remains limited. Therefore, while these studies provide valuable clues, larger, well-characterised multi-ethnic cohort studies, coupled with dedicated functional analyses, are essential to fully elucidate the role of renalase variants in cardiovascular pathophysiology.

## 6. Circulating Renalase as a Potential Biomarker in Cardiovascular and Related Diseases

Beyond genetic predispositions, the measurement of circulating renalase levels (in serum, plasma, or urine) has emerged as an area of significant research interest for its potential utility as a biomarker in various cardiovascular and associated conditions. Fluctuations in renalase concentrations may reflect underlying pathophysiological processes, offer prognostic information, or even guide therapeutic decisions. However, the interpretation of these levels is often complex, influenced by the specific clinical context, patient demographics, comorbidities, and methodologies used for measurement. As will be detailed, the existing literature presents a heterogeneous landscape, with findings often appearing contradictory, a challenge compounded by significant variations in measurement and reporting standards across studies. This section reviews studies that have evaluated renalase as a biomarker across a spectrum of human diseases, highlighting consistencies and discrepancies in the findings (Table 2).

### 6.1. Hypertension and Related Conditions

The association between circulating renalase levels and hypertension presents a particularly intricate picture, with studies reporting varied findings that appear to depend on the specific patient cohort and associated conditions. In adolescent Polish patients with primary hypertension, for example, serum renalase levels were found to be elevated compared to controls and correlated positively with both serum uric acid and blood pressure metrics, suggesting a potential link to early-onset hypertension pathophysiology [66]. Similarly, a long-term study in middle-aged and elderly Chinese hypertensive patients also demonstrated higher serum renalase levels compared to normotensive individuals, with these levels correlating with blood pressure and overall hypertension risk [65].

However, a contrasting pattern emerges when considering specific comorbid conditions like sleep disorders. In middle-aged Polish patients with sleep bruxism, serum renalase levels were lower in the hypertensive subgroup compared to normotensive controls, and these levels negatively correlated with body mass index (BMI) within the bruxism group [67]. This trend of lower renalase in the presence of hypertension and sleep-related disorders was echoed in studies on patients with suspected obstructive sleep apnoea (OSA). One investigation involving middle-aged Polish patients found that renalase levels were lower in individuals with moderate/severe OSA compared to those without OSA, and these levels negatively correlated with the apnoea–hypopnoea index (AHI) in hypertensive individuals, males, and younger subjects [68]. Another study in a similar cohort reported lower renalase levels in the hypertensive group versus those without OSA and noted negative correlations with pulse pressure and echocardiographic parameters like interventricular septum diameter and left atrial diameter [69].

Collectively, these findings suggest that while renalase levels may be elevated in some uncomplicated hypertensive populations, the presence of comorbidities such as sleep-disordered breathing might alter this relationship, often showing an inverse association. The wide variation in reported absolute concentrations across studies (e.g., µg/mL vs. ng/mL) also underscores a potential challenge in direct comparisons, possibly reflecting differences in assay methodologies or patient characteristics that warrant further investigation.

### 6.2. Coronary Artery Disease (CAD)

In the context of CAD, renalase has been explored both as a marker of disease presence or severity and as an indicator of complications related to interventions. For instance, urinary renalase levels have shown promise as a diagnostic marker for contrast-induced acute kidney injury (CI-AKI) following coronary procedures. In elderly Polish CAD patients undergoing coronary angiography/percutaneous coronary interventions (CA/PCI), a significant decrease in the urinary renalase-to-creatinine ratio was observed 6 h post-procedure, irrespective of CI-AKI development. However, a more pronounced decrease (below the 25th percentile) in this ratio was suggested as a specific predictor of CI-AKI [70].

Serum renalase levels have also been linked to the interplay between CAD and CKD. In a cohort of non-diabetic elderly Taiwanese patients with established CAD, those who also had CKD exhibited higher serum renalase and endothelin-1 (ET-1) levels compared to CAD patients without CKD. This study posited that high serum renalase in the presence of CKD might signal an increased risk of elevated ET-1, potentially contributing to atherosclerosis and hypertension [71]. Further highlighting its prognostic relevance in CAD, another study in elderly Taiwanese patients showed that serum renalase levels were significantly reduced after PCI. While pre-PCI brain-derived neurotrophic factor (BDNF) levels predicted this reduction, it was the post-PCI renalase level itself (≥35 ng/mL) that was associated with a higher long-term risk of myocardial infarction, stroke, and death [72]. Beyond macrovascular disease, an association was also identified between higher serum renalase levels and coronary microvascular dysfunction (CMD) in middle-aged American patients with chest pain, suggesting renalase as an independent predictor of CMD, though this requires validation [73].

Overall, these studies suggest multifaceted roles for renalase in CAD, from indicating procedural kidney injury and predicting long-term adverse events post-PCI to associating with microvascular dysfunction and reflecting the burden of comorbid CKD.

### 6.3. Atrial Fibrillation (AF)

The involvement of renalase in atrial fibrillation (AF) has also been investigated, linking its circulating levels to the presence and characteristics of the arrhythmia. In a study of elderly Polish patients with AF undergoing pulmonary vein isolation (PVI), mean serum renalase levels were notably higher compared to a control group. However, within the AF cohort, levels were paradoxically lower in patients with persistent AF (versus non-persistent forms) and in those experiencing AF episodes immediately before PVI. Furthermore, lower renalase levels (specifically, those in the first quartile) were associated with markers of more advanced atrial remodelling and higher AF burden, including increased heart rate, greater AF percentage, larger left atrial (LA) diameter, and a trend towards impaired LA strain. Despite these correlations with arrhythmia burden and structural changes, renalase levels did not predict AF recurrence at a 6-month follow-up in this particular study [74]. These findings suggest a complex, possibly biphasic, relationship between renalase and AF pathophysiology, where renalase may be elevated in the general AF state but reduced in more advanced or active disease manifestations.

### 6.4. Heart Failure (HF)

In patients with heart failure (HF), particularly those with reduced ejection fraction (HFrEF), renalase levels appear to be consistently elevated and associated with disease severity and cardiac remodelling. A series of studies in elderly Serbian HF patients has shed light on this. One investigation found that plasma renalase levels were highest in the HFrEF group compared to those with mid-range (HFmrEF) or preserved ejection fraction (HFpEF), and these levels correlated with left ventricular mass index and plasma B-type natriuretic peptide (BNP) concentrations in HFrEF patients, suggesting a role in predicting left ventricular hypertrophy [75]. Subsequent work by the same research group confirmed higher renalase levels in HFrEF versus HFpEF patients and further demonstrated correlations with a suite of other cardiac remodelling biomarkers (galectin-3, sST2, GDF-15, syndecan-1, BNP, and cystatin), alongside a negative correlation with left ventricular ejection fraction (LVEF) [76]. Expanding on these findings, another study from this group in chronic heart failure (CHF) patients identified that increased levels of renalase, along with other biomarkers like BNP and sST2, in the HFrEF subgroup were independent predictors of ischaemia [77].

Taken together, these consistent findings from a focused research group strongly suggest that elevated plasma renalase levels in HF patients, especially those with HFrEF, are linked to adverse cardiac remodelling, reduced systolic function, and underlying ischaemia, positioning renalase as a potentially valuable biomarker in this specific HF phenotype.

### 6.5. Broader Cardiovascular Risk Factors and Major Adverse Cardiovascular Events (MACEs)

Renalase’s utility as a biomarker extends to broader cardiovascular risk assessment and the prediction of major adverse cardiovascular events (MACEs), particularly in patients with kidney dysfunction. In middle-aged Turkish peritoneal dialysis (PD) patients, median serum renalase levels were higher compared to controls, correlating positively with C-reactive protein (an inflammatory marker) and negatively with residual renal function (RRF), although no direct correlation was found with epicardial adipose tissue (EAT) or left ventricular mass index (LVMI) in this cohort [78].

The prognostic value of renalase in CKD patients has been further highlighted in other studies. An analysis of elderly Polish CKD patients revealed that renalase levels correlated with all-cause death (statistically significant in the haemodialysed subgroup, who had higher levels than controls) and with MACE occurrence in the entire CKD population. Specific cut-off levels (>25 µg/mL for MACE risk; >30 µg/mL for death risk in haemodialysed patients) were proposed [79]. As with other single-centre findings, these prognostic thresholds are preliminary and necessitate validation in external cohorts before clinical consideration. Similarly, research on pre-dialysis elderly Portuguese CKD patients found higher renalase levels in those with more advanced CKD stages. In this population, renalase levels were associated with a wide array of biochemical derangements, eGFR, CKD progression, hospitalisation, and all-cause mortality, although no association with MACE was reported in this particular cohort [80]. These studies collectively underscore renalase’s potential as an indicator of disease severity and adverse outcomes in CKD.

Interestingly, when examining a population with a spectrum of common CVD risk factors (obesity, hypertension, hypercholesterolaemia, diabetes, and others), an inverse relationship was observed. Higher renalase levels were found in a group of middle-aged Polish patients with a lower number of these risk factors, and lower serum renalase was specifically associated with obesity, smoking, and lack of physical activity. A renalase cut-off (<38.98 ng/mL) was even identified as associated with the presence of five or more CVD risk factors [81]. This finding also awaits independent validation. This suggests that in a more general population context, lower renalase might reflect a poorer metabolic and cardiovascular risk profile, contrasting with findings in established, severe disease states like advanced CKD or HFrEF where renalase levels are often elevated.

The body of evidence exploring circulating renalase as a biomarker reveals a complex and often paradoxical picture. A central challenge for interpretation is the heterogeneity of findings, where both elevated and reduced renalase levels have been associated with adverse cardiovascular phenotypes depending on the specific condition. For example, higher renalase is linked to poorer outcomes in HFrEF and advanced CKD, whereas lower renalase is associated with hypertension in the context of sleep apnoea and with a higher burden of general cardiovascular risk factors. This suggests that renalase may not function as a simple “higher is worse” or “lower is worse” biomarker; rather, its interpretation is critically dependent on the clinical context, potentially reflecting different underlying pathophysiological states, such as acute stress response, chronic organ damage, compensatory upregulation, or metabolic dysregulation.

This biological complexity is severely compounded by methodological limitations, as requested for discussion by the reviewers. The vast majority of studies rely on commercial ELISA kits, but there is a profound lack of standardisation in these assays. This leads to wide disparities in reported concentrations, sometimes differing by orders of magnitude (µg/mL vs. ng/mL), making cross-study synthesis nearly impossible. Key unresolved issues include whether different kits detect the same renalase isoforms or post-translationally modified forms, their specificity and cross-reactivity, and the absence of a universal reference standard for calibration. Until the field moves towards assay harmonisation, interpreting and comparing renalase levels across different studies will remain a significant barrier to its clinical validation.

Therefore, while renalase shows context-specific promise, for instance, as a prognostic marker in HFrEF or a predictor of CI-AKI, its utility as a widespread biomarker is currently limited by these unresolved biological and methodological inconsistencies. Future research, as will be outlined in the Discussion, must focus on addressing these critical gaps to harness its potential.

## 7. Discussion

**Figure 3:**
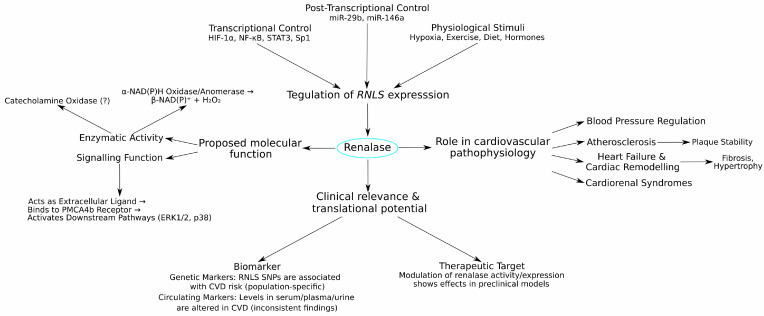
Conceptual framework of the multifaceted roles of renalase in cardiovascular disease. This diagram provides an integrated overview of renalase, showing the factors that regulate its production, its diverse proposed functions, its involvement in key cardiovascular pathologies, and its translational potential. **(1) Regulation of** ***RNLS*** **Expression:** The production and levels of renalase are controlled by a complex network of inputs. These include transcriptional control by factors such as HIF-1α, NF-κB, and STAT3; post-transcriptional control by microRNAs (e.g., miR-29b, miR-146a); and physiological stimuli like hypoxia, exercise, and diet. **(2) Proposed Molecular Functions:** Once produced, renalase is proposed to have multiple functions. Its enzymatic activity is debated, with proposed roles as a catecholamine oxidase (controversial) and an α-NAD(P)H oxidase/anomerase that generates β-NAD(P)^+^ and H_2_O_2_. Additionally, it has a signalling function, acting as an extracellular cytokine-like ligand that binds to the PMCA4b receptor to activate downstream pathways such as ERK1/2 and p38. **(3) Role in Cardiovascular Pathophysiology:** Through its functions, renalase is implicated in several key disease processes, including blood pressure regulation, atherosclerosis (influencing plaque stability), heart failure, and cardiac remodelling (modulating fibrosis and hypertrophy). **(4) Clinical Relevance & Translational Potential:** The involvement of renalase in CVD makes it clinically relevant. It is studied as a biomarker, both through genetic variations in the *RNLS* gene (SNPs) and as a circulating protein whose levels are altered in disease. It is also explored as a potential therapeutic target, where preclinical models suggest that modulating its activity or expression could have beneficial effects. Abbreviations: CVD, cardiovascular disease; ERK1/2, Extracellular signal-regulated kinase 1/2; H_2_O_2_, hydrogen peroxide; HIF-1α, Hypoxia-Inducible Factor-1α; miR, microRNA; NF-κB, Nuclear Factor kappa B; PMCA4b, Plasma Membrane Ca^2+^-ATPase isoform 4b; *RNLS*, renalase gene; SNP, Single Nucleotide Polymorphism; STAT3, Signal Transducer and Activator of Transcription 3.

The scientific journey of renalase, from its initial characterisation as a putative FAD-dependent amine oxidase to its current depiction as a multifaceted protein with potential enzymatic and signalling roles, underscores its complex biology. This review has charted its discovery, intricate gene regulation, involvement in preclinical cardiovascular models, diverse genetic associations in human populations, and its varied profile as a clinical biomarker. While the originally proposed primary function in catecholamine degradation and direct blood pressure control has been widely debated and often challenged, alternative functions, including α-NAD(P)H oxidase/anomerase activity and cytokine-like signalling through the PMCA4b receptor, offer new avenues for understanding its physiological and pathological significance. As conceptualised in Figure 3, renalase sits at a nexus of metabolic, signalling, and stress–response pathways relevant to cardiovascular health.

### 7.1. Renalase in Specific Cardiovascular Contexts: Synthesis and Critical Appraisal

• **Hypertension and Related Syndromes:**

The relationship between renalase and hypertension appears particularly nuanced. While some studies, especially in younger or uncomplicated hypertensive cohorts, report *elevated* circulating renalase levels that correlate with blood pressure, findings in the presence of comorbidities such as obstructive sleep apnea or sleep bruxism often show an inverse relationship, with *lower* renalase levels in hypertensive individuals. This discrepancy suggests that the interplay between renalase and blood pressure regulation is highly context-dependent, possibly influenced by factors like age, underlying sympathetic tone alterations in sleep disorders, and interactions with other neurohumoral systems. Genetic studies also point to certain *RNLS* polymorphisms being associated with hypertension risk or salt sensitivity of blood pressure, but these associations often exhibit population-specificity, indicating complex gene–environment interactions. The broad range of reported renalase concentrations (from ng/mL to µg/mL) in these studies further complicates a unified interpretation, highlighting a significant methodological challenge.

• **CAD and Atherosclerosis:**

In the realm of CAD and atherosclerosis, renalase has emerged in several distinct contexts. Preclinical work in *ApoE^−/−^* mice suggests a potential role in plaque stabilisation, possibly modulated by therapeutic agents like valsartan. In human CAD patients, urinary renalase shows promise as a marker for contrast-induced acute kidney injury post-intervention. Serum renalase levels have been linked to the burden of comorbid chronic kidney disease in CAD patients and, perhaps more critically, post-PCI renalase levels have been identified as a potential long-term prognostic marker for MACE. Furthermore, elevated renalase has been associated with coronary microvascular dysfunction. These diverse findings suggest renalase might reflect different aspects of CAD pathophysiology—from endothelial dysfunction and inflammatory responses to the impact of renal impairment and procedural stress. Again, the variability in measured levels and the specific renalase species (e.g., urinary vs. serum) assessed are critical considerations.

• **Heart Failure (HF) and Cardiac Remodelling:**

Preclinical models of pressure overload-induced LVH and CKD-induced cardiac remodelling consistently implicate renalase in the processes of cardiac hypertrophy and fibrosis, with its modulation (either genetically or via recombinant protein) affecting key signalling pathways like p38 and ERK1/2, and altering ECM deposition. These findings align well with human studies, particularly in patients with heart failure with reduced ejection fraction (HFrEF), where elevated plasma renalase levels are consistently reported and correlate with markers of adverse cardiac remodelling, reduced systolic function (LVEF), and ischaemia. This relatively consistent picture in HFrEF suggests that renalase might be a more robust biomarker in this specific HF phenotype, potentially reflecting the degree of myocardial stress or dysfunction.

• **Atrial Fibrillation (AF):**

The association of renalase with atrial fibrillation presents a more intricate pattern. While overall renalase levels may be higher in AF patients undergoing PVI compared to controls, they appear to be paradoxically lower in those with persistent AF or active pre-procedural episodes. Moreover, lower renalase has been linked to markers of more advanced atrial structural and electrical remodelling. This could imply a biphasic role, where renalase initially increases as part of a stress response but may become depleted or downregulated in more chronic or severe states of atrial disease. The lack of predictive value for AF recurrence in one study, despite these associations, suggests that its role as a simple prognostic marker in AF needs further clarification.

• **Chronic Kidney Disease (CKD) and Broader Cardiovascular Risk:**

In CKD, particularly in advanced stages or in patients undergoing dialysis, circulating renalase levels are often significantly elevated and correlate with disease severity, inflammation, and adverse outcomes, including all-cause mortality and MACE (in some studies). This strongly suggests that renalase metabolism or clearance is impaired in CKD, or that it is upregulated as part of the uraemic state or systemic inflammation. In contrast, when examining populations with an accumulation of general cardiovascular risk factors (but not necessarily advanced organ damage), some evidence points towards *lower* renalase levels being associated with a poorer metabolic profile and a higher number of risk factors. This apparent contradiction highlights that renalase’s interpretation may drastically differ depending on whether it’s viewed in the context of early risk accumulation versus established end-organ damage.

### 7.2. Key Challenges and Limitations in Current Renalase Research

**Figure 4:**
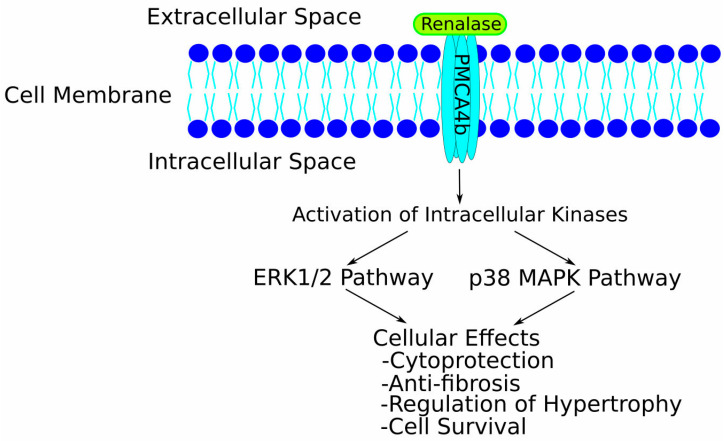
Proposed signalling pathway of extracellular renalase. Extracellular renalase functions as a cytokine-like ligand by binding to its specific cell surface receptor, the plasma membrane Ca^2+^-ATPase isoform PMCA4b. This binding event triggers the activation of downstream intracellular signalling cascades, including the ERK1/2 and p38 MAPK pathways. These pathways, in turn, mediate many of renalase’s biological effects, such as cytoprotection, anti-fibrotic actions, and the regulation of cellular hypertrophy and survival.

The translation of renalase research into robust clinical applications is currently hampered by several key challenges and limitations inherent in the existing body of literature:

• **Methodological Heterogeneity in Renalase Quantification:** A primary obstacle is the profound lack of standardisation in measuring renalase. Studies employ different ELISA kits, report values in widely disparate units (µg/mL and ng/mL), assess different biofluids (serum, plasma, urine), and may not distinguish between total and potentially active renalase. This makes direct comparisons of absolute values across studies nearly impossible and contributes significantly to perceived inconsistencies in findings. Specific limitations of ELISA-based methods include potential variability in antibody specificity and affinity for different renalase isoforms or post-translationally modified forms, a lack of universally accepted calibrators, and significant inter-laboratory variation. Without a harmonised approach, the field cannot establish reliable reference ranges or validated prognostic cut-offs.
• **Inconsistency and Context-Dependency of Findings:** As highlighted above, the direction and strength of association between renalase (levels or SNPs) and clinical parameters often vary considerably depending on the specific disease, its stage, patient demographics (age, sex, ethnicity), comorbidities, and even medication use. This suggests that renalase is not a “one-size-fits-all” biomarker and its interpretation requires careful contextualisation.
• **Discerning Causation from Correlation:** Many human studies are cross-sectional or observational, establishing associations rather than causal relationships. It is often unclear whether altered renalase levels are a pathogenic driver, a compensatory mechanism, or simply an epiphenomenon of the underlying disease state.
• **Functional Impact of Genetic Variants:** While numerous *RNLS* SNPs have been linked to CVD risk, the functional consequences of most of these variants on renalase expression, protein stability, or enzymatic/signalling activity remain largely uncharacterised.
• **Uncertainty Regarding Primary Enzymatic Functions:** A critical challenge remains the unresolved debate over renalase’s primary enzymatic function in vivo. While the α-NAD(P)H oxidase activity provides a plausible biochemical role with clear implications for cellular redox balance, the identification of a specific cell surface receptor, PMCA4b, lends strong, independent support to its function as an extracellular signalling molecule, acting akin to a cytokine (as illustrated in Figure 4). These roles are not mutually exclusive; renalase may function differently inside the cell versus in circulation. However, the lack of definitive in vivo evidence for either catecholamine degradation or significant NAD(P)H isomer oxidation in circulation complicates the interpretation of its physiological effects.

### 7.3. Future Directions and Opportunities

To overcome these limitations and fully realise the potential of renalase in cardiovascular medicine, future research should prioritise several key areas:

• **Standardisation and Methodological Advancements:**
  • Develop and validate standardised, robust, and widely accessible assays for quantifying renalase levels and, if possible, its specific enzymatic activity in various biological samples. Establishing reference materials and agreed-upon reporting units is critical.
  • Further refine techniques to differentiate between various renalase isoforms and post-translationally modified forms that may have distinct biological activities.
• **Elucidating Fundamental Biology and Mechanisms:**
  • Conduct definitive studies to identify the primary physiological substrates and products of renalase in vivo under varying conditions.
  • Deepen the understanding of renalase signalling pathways, including receptor interactions beyond PMCA4b and downstream intracellular events (see Figure 4 for the current model).
  • Perform comprehensive functional characterisation of disease-associated RNLS SNPs to determine their impact on gene expression, protein function, and ultimately, cellular and organismal phenotypes.
• **Preclinical and Translational Research:**
  • Utilise advanced preclinical models (e.g., tissue-specific conditional knockouts/ins, humanised mice) to dissect the context-specific roles of renalase in different organs and cell types during CVD development and progression.
  • Investigate the therapeutic potential of modulating renalase (e.g., via recombinant protein, gene therapy, or pharmacological agents) in well-defined disease models, with clear mechanistic readouts.
• **Robust Human Clinical Studies:**
  • Design and execute large-scale, prospective, multi-ethnic cohort studies to rigorously assess the diagnostic, prognostic, and predictive value of renalase (levels and SNPs) for specific cardiovascular outcomes, using the standardised assays developed above.
  • Explore the utility of renalase in combination with other biomarkers to enhance predictive accuracy for complex cardiovascular phenotypes.
  • Crucially, for any proposed clinical application, studies must address practical considerations such as assay cost-effectiveness, turnaround time, and potential integration into existing clinical guidelines.

## 8. Summary and Conclusions: Renalase in the Cardiovascular Nexus—Current Standing and Future Horizons

Having navigated the intricate landscape of renalase research, from its molecular underpinnings and complex regulatory networks to its diverse manifestations in preclinical models and human cardiovascular disease, a salient picture emerges: renalase is a multifaceted protein whose full significance in cardiovascular health and pathology is still being actively deciphered. This review has traversed nearly two decades of scientific inquiry, revealing a molecule initially conceived with a relatively narrow function that has since expanded to encompass a wide spectrum of potential enzymatic and signalling roles, deeply entwining it with cellular metabolism, stress responses, and intercellular communication.

The journey has underscored a critical shift from the early, debated hypothesis of renalase as primarily a catecholamine-degrading enzyme pivotal for blood pressure homeostasis. While this initial concept spurred much research, the collective evidence now points towards a more nuanced reality, suggesting that if renalase does interact with catecholamines, this is likely one facet of a broader functional profile. The compelling, albeit still developing, evidence for its α-NAD(P)H oxidase/anomerase activity and its capacity to act as a secreted signalling ligand through receptors such as PMCA4b suggests that renalase operates at multiple levels, potentially influencing intracellular redox balance, energy metabolism, and cellular protective or pro-pathogenic signalling cascades.

This inherent functional versatility is mirrored by the complexity of its gene regulation, which is responsive to an array of transcriptional, post-transcriptional, and physiological modulators, leading to context-dependent expression and activity. Such elaborate control likely underpins the often varied observations in preclinical cardiovascular disease models, where renalase manipulation can yield diverse, sometimes protective, sometimes detrimental outcomes in conditions like atherosclerosis and heart failure, contingent on the specific model, timing, and nature of the intervention.

In the human arena, the study of *RNLS* gene polymorphisms has revealed numerous statistical associations with cardiovascular traits and disease susceptibilities. However, the clinical translation of these genetic findings is tempered by their frequent population-specificity and the general lack of direct functional evidence linking specific variants to altered renalase biology in a disease-relevant manner. The exploration of circulating renalase as a clinical biomarker has perhaps generated the most extensive, yet also the most heterogeneous, body of data. While intriguing correlations with disease presence, severity, and prognosis have been reported across a wide range of cardiovascular conditions, such as hypertension and coronary artery disease, to atrial fibrillation and heart failure, the notable inconsistencies in findings and the profound lack of methodological standardisation in renalase quantification present formidable challenges to its widespread clinical adoption. It is clear that factors such as disease stage, comorbidities, patient demographics, and the specific biofluid and assay employed profoundly influence measured renalase levels, necessitating highly contextualised interpretation.

In synthesising this expansive body of work, renalase emerges not as a simple biomarker or a straightforward therapeutic target, but as an integral component of a complex biological network whose precise contributions to cardiovascular health and disease are conditional and context-specific. Its true clinical utility is unlikely to be as a standalone, universal marker, but perhaps as part of a multi-marker panel or in highly specific clinical scenarios. Future progress in the field will be critically dependent on resolving fundamental questions about its primary in vivo functions, achieving robust standardisation of its measurement, and conducting large-scale, mechanistically informed clinical studies. Only through such concerted efforts can the “renalase enigma” be unpuzzled, allowing the scientific and medical communities to definitively harness its potential for improving the diagnosis, prevention, and treatment of cardiovascular diseases.

## Figures and Tables

**Table 1:** Associations of Renalase Gene Variants with Cardiovascular Diseases and Related Phenotypes.

SNP(s)	Patient Cohort Characteristics	Key Findings	References
rs2296545 (Glu37Asp)	657 elderly Polish patients with aortic stenosis	Asp37 variant associated with higher left ventricular mass, intraventricular septal thickness, posterior wall thickness, and relative wall thickness in female patients only; no association in male patients.	[57]
	126 middle-aged Chinese Han patients with OSA	Serum renalase levels higher in severe OSA group; positive correlation with BP in non-OSA group, negative correlation in severe OSA group. CG genotype associated with increased hypertension risk in the severe OSA group.	[58]
	60 middle-aged Egyptian patients with CKD (hypertensive and normotensive)	CC genotype and C allele associated with increased risk of CKD and higher risk of developing hypertensive CKD.	[59]
rs10887800	260 elderly Iranian patients with CAD and hypertension	Renalase activity higher in hypertension patients. No gender differences in renalase activity. No association of SNP with risk/protection against hypertension and/or CAD. Atorvastatin/losartan increased renalase activity (hypertension); losartan reduced renalase activity (CAD) vs. controls.	[60]
	134 elderly Iranian patients with MetS and USAP	Renalase levels higher in USAP + MetS patients (23.28 ± 4.09 µg/dL) vs. healthy controls (20.81 ± 2.73 µg/dL). AG and GG genotypes more common in USAP + MetS patients.	[61]
rs2576178 rs10887800	154 middle-aged Iranian patients with IS	rs10887800 AG genotype associated with a 1.6-fold higher risk of IS. No significant association for rs2576178.	[62]
rs2576178	449 elderly Chinese CAD patients	GG genotype or G allele associated with higher CAD risk in females, smokers, and individuals who consume alcohol. No association with general clinical parameters.	[63]
rs1932531, rs77324767, rs10749565, JHU_10.89888367, rs77287889, rs868872, rs10509536; (TCTTAGTT haplotype) rs10887801	720 middle-aged American patients with hypertension	On high-salt diet, 7 SNPs (TCTTAGTT haplotype) associated with SBP, pulse pressure, and mean arterial pressure. rs10887801 TT genotype carriers had higher SSBP and lower plasma renin activity.	[64]
rs7922058, rs1935582, rs2576178, rs10887800, rs796945, rs2296545	488 middle-aged and elderly Chinese hypertensive patients	rs7922058: associated with 14-year change in systolic BP. rs1935582 & rs2576178: associated with 14-year change in mean arterial pressure. rs2576178, rs1935582, rs10887800, rs796945, & rs2296545: associated with 14-year change in diastolic BP. rs1935582, rs2576178, & rs796945: associated with hypertension incidence. Renalase gene expression decreased in renal biopsies of hypertensive vs. normotensive controls.	[65]

Abbreviations: BP, blood pressure; CAD, coronary artery disease; CKD, chronic kidney disease; IS, ischaemic stroke; MetS, metabolic syndrome; OSA, obstructive sleep apnoea; SBP, systolic blood pressure; SSBP, salt sensitivity of blood pressure; USAP, unstable angina pectoris.

**Table 2:** Circulating Renalase Levels and Their Associations in Various Human Cardiovascular and Related Conditions.

Condition Studied/Main Grouping	Patient/Cohort Description	*n*	Renalase Levels (Mean ± SD or Median [IQR/Range]) & Units	Key Associations and Findings	References
**Hypertension & Related** **Conditions**					
Primary Hypertension	Polish adolescent patients	88	Serum: Patients: Med 29.8 [26.1–35.8] µg/mL Controls: Med 26.8 [22.96–29.4] µg/mL	Higher in patients. Correlated with serum uric acid. Correlated with systolic & diastolic BP in hypertensive patients.	[66]
Sleep Bruxism (Hypertensive vs. Normotensive)	Polish middle-aged patients	87	Serum: Hypertensive: 133.33 ± 160.71 ng/mL Normotensive Controls: 219.23 ± 220.58 ng/mL	Lower in hypertensive group. Negatively correlated with BMI (bruxism group). Cut-off > 212.5 ng/mL associated higher bruxism episodes & BMI with lower renalase.	[67]
Suspected Obstructive Sleep Apnoea (OSA)	Polish middle-aged patients	113	Serum: Mod/Sev OSA: 139.56 ± 175.72 ng/mL No OSA (Controls): 230.97 ± 240.50 ng/mL	Lower in moderate/severe OSA. Negatively correlated with AHI (hypertensive group, males, <60 yrs). Lower renalase, hypertension, higher BMI, male gender independently associated with higher AHI.	[68]
	Polish middle-aged patients	101	Serum: Hypertensive: 159.16 ± 207.19 ng/mL No OSA (Controls): 212.56 ± 238.79 ng/mL OSA group (general): 167.37 ± 197.29 ng/mL	Negatively correlated with pulse pressure, IVSEDD, & LA diameter. Lower in hypertensive group vs. no OSA; no significant difference in general OSA group vs. no OSA.	[69]
Hypertension	Chinese middle-aged & elderly patients	488	Serum: Hypertensive: 27.2 ± 0.4 µg/mL Normotensive: 25.1 ± 0.2 µg/mL	Higher in hypertensive patients. Correlated with BP and associated with hypertension risk.	[65]
**Coronary Artery Disease (CAD)**					
CI-AKI post-CA/PCI	Polish elderly patients with CAD	95	**Urinary** (Renalase/Creatinine ratio): Pre-CA/PCI: 2843.6 ng/mg 6 h Post-CA/PCI: 1540.7 ng/mg (Decreased in both CI-AKI & non-CI-AKI subgroups)	**Urinary** ratio decreased 6 h post-CA/PCI. Decrease below 25th percentile suggested as CI-AKI predictor.	[70]
CAD with/without CKD	Non-diabetic elderly Taiwanese patients	342	Serum: CAD + CKD: 46.8 ± 17.1 ng/mL CAD (no CKD): 33.9 ± 9.9 ng/mL	Higher renalase (and ET-1) in CAD + CKD. High renalase with CKD associated with increased ET-1.	[71]
Post-PCI Outcomes	Taiwanese elderly CAD patients	152	Serum: Pre-PCI: 47.5 ± 17.3 ng/mL Post-PCI: 35.9 ± 11.3 ng/mL	Reduced post-PCI. Pre-PCI BDNF predicted renalase reduction. Post-PCI renalase ≥ 35 ng/mL associated with higher risk of MI, stroke, death.	[72]
Coronary Microvascular Dysfunction (CMD)	American middle-aged patients with chest pain (PET/CT)	80	Serum: CMD: Med 5503 [IQR 3070] ng/mL CAD: Med 4069 [IQR 1850] ng/mL Controls: Med 4266 [IQR 1503] ng/mL	Higher in CMD patients. Independent predictor of CMD.	[73]
**Atrial Fibrillation (AF)**					
AF undergoing PVI	Polish elderly patients	69	Serum: AF (PVI): Mean 27.99 µg/mL Controls: Mean 21.48 µg/mL Persistent AF: 19.05 µg/mL (vs. 28.77 µg/mL non-persistent)	Higher in AF (PVI) vs. controls. Lower in persistent AF & with pre-PVI AF episodes. Lower levels associated with higher heart rate, AF burden, LA diameter, less negative LA strain. No prediction of AF recurrence (6 mo).	[74]
**Heart Failure (HF)**					
HF (HFrEF, HFmrEF, HFpEF)	Serbian elderly patients	75	Plasma: HFrEF: 147.33 ± 29.07 ng/mL HFmrEF: 118.58 ± 19.61 ng/mL HFpEF: 117.31 ± 32.83 ng/mL	Higher in HFrEF. Correlated with LV mass index & BNP in HFrEF.	[75]
HF (HFrEF vs. HFpEF)	Serbian elderly patients	76	Plasma: HFrEF: 126.26 ± 8.45 ng/mL HFpEF: 106.68 ± 13.54 ng/mL	Higher in HFrEF. Correlated with remodelling biomarkers (galectin-3, sST2, GDF-15, syndecan-1, BNP, cystatin); negatively with LVEF.	[76]
Chronic HF (CHF)	Serbian elderly patients	77	Plasma: HFrEF: 147.52 ± 29.39 ng/mL HFpEF: 122.63 ± 38.61 ng/mL	Increased renalase (and other biomarkers) in HFrEF were independent predictors of ischaemia.	[77]
**CVD Risk Factors/MACE/CKD**					
Peritoneal Dialysis (PD)	Turkish middle-aged patients	40	Serum: PD: Med 176.5 [100–278.3] ng/mL Controls: Med 122 [53.3–170.0] ng/mL	Higher in PD patients. Correlated with CRP, negatively with RRF. No correlation with EAT or LVMI.	[78]
Chronic Kidney Disease (CKD)	Polish elderly patients (incl. haemodialysed)	90	Serum: Haemodialysed CKD: 35.6 ± 13.5 µg/mL Controls: 21.8 ± 9.2 µg/mL	Correlated with all-cause death (haemodialysed subgroup). Correlated with MACE & all-cause death (entire CKD pop.). Cut-offs: >25 µg/mL (MACE risk); >30 µg/mL (death risk in haemodialysed).	[79]
Pre-dialysis CKD	Portuguese elderly patients	40	Serum: CKD Stage 4–5: Med 83.53 [78.6–95.2] µg/mL CKD Stage 1–3: Med 42.03 [37.45–60.07] µg/mL	Higher in advanced CKD stages. Associated with various biochemical parameters, eGFR, CKD progression, hospitalisation, all-cause mortality. Negatively correlated with Hb, HDL. No MACE association reported.	[80]
Multiple CVD Risk Factors	Polish middle-aged patients with diverse risk factors	96	Serum: Lower RF group: 284.33 ± 232.20 ng/mL Higher RF group (≥4 RFs): 90.33 ± 127.50 ng/mL	Higher renalase with fewer CVD risk factors. Lower renalase associated with obesity, smoking, lack of physical activity. Cut-off < 38.98 ng/mL associated with ≥5 CVD risk factors.	[81]

Abbreviations: AF, atrial fibrillation; AHI, apnoea–hypopnoea index; BDNF, brain-derived neurotrophic factor; BMI, body mass index; BNP, B-type natriuretic peptide; BP, blood pressure; CA/PCI, coronary angiography/percutaneous coronary interventions; CAD, coronary artery disease; CHF, chronic heart failure; CI-AKI, contrast-induced acute kidney injury; CKD, chronic kidney disease; CMD, coronary microvascular dysfunction; CRP, C-reactive protein; EAT, epicardial adipose tissue; eGFR, estimated glomerular filtration rate; ET-1, endothelin-1; GDF-15, growth differentiation factor 15; Hb, haemoglobin; HDL, high-density lipoprotein; HF, heart failure; HFmrEF, heart failure with mid-range ejection fraction; HFpEF, heart failure with preserved ejection fraction; HFrEF, heart failure with reduced ejection fraction; IQR, interquartile range; IVSEDD, interventricular septum end-diastolic diameter; LA, left atrium/atrial; LV, left ventricular; LVEF, left ventricular ejection fraction; LVMI, left ventricular mass index; MACE, major adverse cardiovascular events; Med, median; MI, myocardial infarction; Mod/Sev, moderate/severe; OSA, obstructive sleep apnoea; PD, peritoneal dialysis; PET/CT, positron emission tomography/computed tomography; PVI, pulmonary vein isolation; RF, risk factor(s); RRF, residual renal function; sST2, soluble suppression of tumourigenicity 2; vs., versus.

## Data Availability

Not applicable.
